# Tumor Cellularity Assessment of Breast Histopathological Slides via Instance Segmentation and Pathomic Features Explainability

**DOI:** 10.3390/bioengineering10040396

**Published:** 2023-03-23

**Authors:** Nicola Altini, Emilia Puro, Maria Giovanna Taccogna, Francescomaria Marino, Simona De Summa, Concetta Saponaro, Eliseo Mattioli, Francesco Alfredo Zito, Vitoantonio Bevilacqua

**Affiliations:** 1 Department of Electrical and Information Engineering (DEI), Polytechnic University of Bari, Via Edoardo Orabona n. 4, 70126 Bari, Italy; 2 Molecular Diagnostics and Pharmacogenetics Unit, IRCCS Istituto Tumori “Giovanni Paolo II”, Via O. Flacco n. 65, 70124 Bari, Italy; 3 Laboratory of Preclinical and Translational Research, Centro di Riferimento Oncologico della Basilicata (IRCCS-CROB), Via Padre Pio n. 1, 85028 Rionero in Vulture, Italy; 4 Pathology Department, IRCCS Istituto Tumori “Giovanni Paolo II”, Via O. Flacco n. 65, 70124 Bari, Italy; 5 Apulian Bioengineering s.r.l., Via delle Violette n. 14, 70026 Modugno, Italy

**Keywords:** nuclei detection, tumor cellularity, instance segmentation, explainable artificial intelligence

## Abstract

The segmentation and classification of cell nuclei are pivotal steps in the pipelines for the analysis of bioimages. Deep learning (DL) approaches are leading the digital pathology field in the context of nuclei detection and classification. Nevertheless, the features that are exploited by DL models to make their predictions are difficult to interpret, hindering the deployment of such methods in clinical practice. On the other hand, pathomic features can be linked to an easier description of the characteristics exploited by the classifiers for making the final predictions. Thus, in this work, we developed an explainable computer-aided diagnosis (CAD) system that can be used to support pathologists in the evaluation of tumor cellularity in breast histopathological slides. In particular, we compared an end-to-end DL approach that exploits the Mask R-CNN instance segmentation architecture with a two steps pipeline, where the features are extracted while considering the morphological and textural characteristics of the cell nuclei. Classifiers that are based on support vector machines and artificial neural networks are trained on top of these features in order to discriminate between tumor and non-tumor nuclei. Afterwards, the SHAP (Shapley additive explanations) explainable artificial intelligence technique was employed to perform a feature importance analysis, which led to an understanding of the features processed by the machine learning models for making their decisions. An expert pathologist validated the employed feature set, corroborating the clinical usability of the model. Even though the models resulting from the two-stage pipeline are slightly less accurate than those of the end-to-end approach, the interpretability of their features is clearer and may help build trust for pathologists to adopt artificial intelligence-based CAD systems in their clinical workflow. To further show the validity of the proposed approach, it has been tested on an external validation dataset, which was collected from IRCCS Istituto Tumori “Giovanni Paolo II” and made publicly available to ease research concerning the quantification of tumor cellularity.

## 1. Introduction

In modern clinical practice, digital pathology covers a crucial role and has increasingly become an indispensable technological requirement in biomedical scientific laboratories. Technological advancements and a greater emphasis on precision medicine have recently paved the way for the development of digital tools based on whole slide imaging and artificial intelligence (AI), which would allow for the analysis of features beyond visual human perception. One of the main applications of digital pathology consists in the classification of cell nuclei for the diagnosis of complex pathologies such as breast cancer, which is the most common form of cancer among women. Currently, cancer diagnosis occurs through the careful analysis of pathologists, who manually examine tissue biopsy samples; it is a laborious and time-consuming procedure that is affected by inter- and intra-operator variability. The development of high-resolution whole slide image (WSI) scanners has enabled the realization of algorithms that automatically perform an accurate and efficient histopathological diagnosis, alleviating the global shortage of trained pathologists [1].

To solve the challenge of the nuclei classification of breast cancer images, various techniques are used that belong to the machine learning (ML) and deep learning (DL) families [2]. To properly classify cell nuclei, the first step involves the precise detection of nuclear boundaries. Then, pathomic features can be extracted from the region of interest, providing quantitative information about the nuclear size, shape, texture, color, and so on [3]. The decision concerning which features are more discriminative for the task is not easy, and it heavily depends on the specific domain. To address these problems, automatic feature representation learning using deep neural networks has become an attractive and popular alternative. In deep learning models, the neural network can be treated as a feature extractor that can obtain hierarchical representations of the data, automatically learning the strongest features needed for the classification [4]. On the other hand, these features are not easy to explain. Indeed, many recent studies in the literature concerning deep learning for medical image analyses have considered explainable artificial intelligence (XAI) methodologies [5,6,7,8]. Furthermore, the explainability of the features is also important from a legal perspective. In fact, the EU General Data Protection Regulation (GDPR), with Articles 13 and 14, decrees that when data subjects are profiled (this concept also applies to the medical field), they have a right to “meaningful information about the logic involved” [9]. Hence, there is a need for explainable computer-aided diagnosis (CAD) systems in digital pathology.

In this work, an explainable workflow for detecting, segmenting, and classifying nuclei from histopathological images stained with hematoxylin and eosin (H&E) is presented. In detail, we exploited two datasets, one of which is the largest publicly available nuclei-annotated dataset of breast cancer slides, whereas the other is a locally collected dataset from IRCCS Istituto Tumori “Giovanni Paolo II”; the two datasets were exploited in order to compare two different strategies. The first one involves the end-to-end adoption of two-class instance segmentation methods, whereas the second one consists of two steps: (i) nuclei detection with single-class instance segmentation; and (ii) tumor/non-tumor classification from pathomic features. In addition, an approach based on SHAP (Shapley additive explanations) [10] has been implemented to explain the output of the machine learning model, which allowed us to focus on the most important classification features. Finally, a collaborative workflow between pathologists and AI models is proposed to evaluate breast cancer samples by integrating obtained results with QuPath [11], a cross-platform, user-friendly, open-source software for digital pathology and WSI analysis.

In summary, our paper introduces the following innovative contributions: (a) the introduction of a tool that can aid pathologists in performing the quantitative assessment of tumor cellularity from histopathological images; (b) the explainability of pathomic features as considered by an ML classifier for distinguishing tumor nuclei, which was validated by an expert pathologist; and (c) the collection of a new dataset of whole slide images from patients with breast cancer from IRCCS Istituto Tumori “Giovanni Paolo II”, which has been made publicly available [12] to ease the development of CAD systems in tumor cellularity assessment.

The remainder of this paper is organized as follows. In Section 2, we describe the datasets adopted and the different methodologies employed to enable the proposed pipeline, including its integration with the QuPath software; focus is ensured on the SHAP method that we employed for the explainability of nuclear features. Section 3 shows the results obtained with the two different approaches and the findings from the SHAP analysis, which are then discussed in Section 4; specifically, Section 4.2 provides a clinical perspective on the considered feature set, validating the analysis through the assessment of an expert pathologist. Finally, Section 5 concludes the paper and offers perspectives for future works.

## 2. Materials and Methods

### 2.1. Datasets

To realize the experiments described in the next subsections, we considered two datasets. In detail, we exploited a publicly available dataset [13] to perform the training and internal validation phases of the considered ML and DL models. Then, a local dataset was collected from the Pathology Department of IRCCS Istituto Tumori “Giovanni Paolo II” to perform an external validation and proof of integration with the QuPath platform. In order to ease the development of CAD systems for the assessment of tumor cellularity in breast cancer, we made our dataset publicly available [12].

**NuCLS**—The NuCLS dataset [13] contains whole slide scans of H&E slides of breast carcinomas from 18 institutions from The Cancer Genome Atlas (TCGA) [14], which were acquired using a magnification factor of 40×. The annotations of each cell nucleus are provided by pathologists, domain experts, and medical students. We only used the corrected single-rater dataset, which were split into the training (1481 images) and validation sets (263 images) as described by the original authors. Annotations for the corrected single-rater dataset, organized in a CSV file with WSI-related coordinates at the basic resolution, included 59,485 nuclei and 19,680 boundaries extracted from 1744 H&E images of variable dimensions, which ranged between 200 and 400 pixels. These images are regions of interest (ROIs) that are selected within each slide by the study coordinator and are representative of the classes and textures of the predominant region. In fact, the annotations include a total of 5 super-classes: *Tumor*, *Stromal*, *sTILs* (stromal tumor-infiltrating lymphocytes), *Other*, and *Ambiguous*. The further subdivisions within the super-classes have not been considered for the study. Because we were interested in the classification of tumor nuclei, all the other super-classes have been fused into a single non-tumor class.

**Local dataset**—The images used for the validation phase belong to a local dataset collected from the Department of Pathology at the IRCCS Istituto Tumori “Giovanni Paolo II” [12]. The dataset consists of 2 H&E whole slide images obtained from patients with breast cancer, captured at a magnification of 40×, and contains an overall amount of tens of thousands of tumor nuclei. The annotations, which consist of tumor and non-tumor nuclei, were provided by an expert pathologist using the QuPath software. In detail, within the indicated ROIs, 1419 tumor nuclei and 605 non-tumor nuclei were labeled for a total of 2024 nuclei (respectively, 762 and 381 from the first WSI, 657 and 224 from the second WSI).

**Figure 1:**
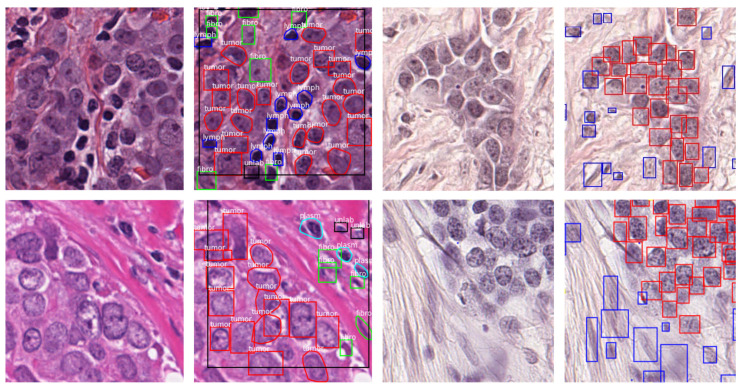
Sample images and annotations of the publicly available and local datasets. (**First Column**) Sample image of the publicly available dataset. (**Second Column**) Annotations as reported in the image text. (**Third Column**) Sample image of the local dataset. (**Fourth Column**) Annotations: red represents tumor nuclei; blue represents non-tumor nuclei.

Sample images of both datasets are portrayed in Figure 1, whereas Table 1 summarizes relevant information for the considered datasets.

### 2.2. Proposed Workflow

The overall workflow comprises several steps. First, the operator selects a relevant ROI on the WSI using the QuPath interface. Then, the designated ROI is tiled into smaller chunks (in this study, we used images with a size of 256 × 256 and an overlap of 128 pixels), which are fed to a detection algorithm. The results are collected tile-by-tile, and then, the whole prediction is reconstructed and portrayed on the QuPath interface for being visualized by the medical experts.

**Figure 2:**
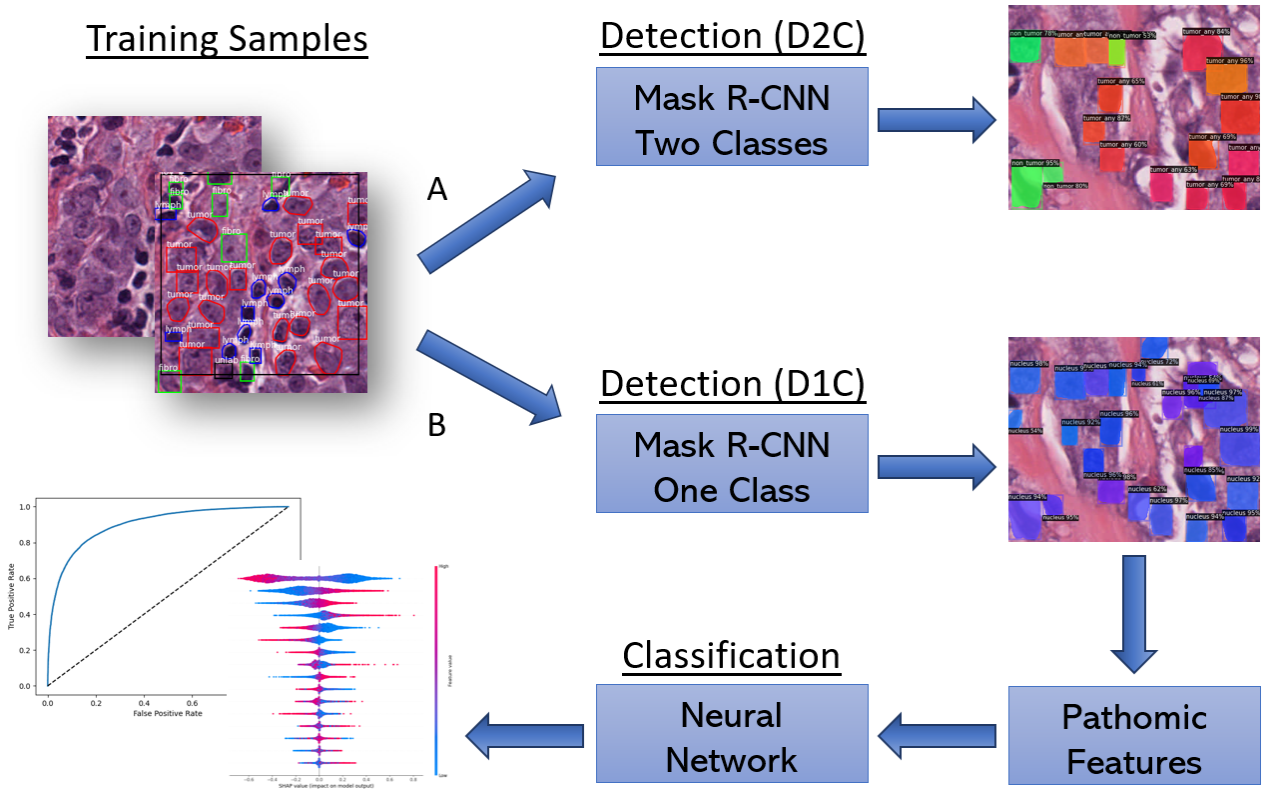
Study workflow (**A**: end-to-end approach, **B**: two-stage approach). The end-to-end approach exploits the Mask R-CNN method with two classes to perform both the detection and classification of tumor and non-tumor nuclei. The two-stage approach, on the other hand, permits us to focus on pathomic features that can be used to gain an understanding of which textural and morphological characteristics are more relevant for recognizing tumor nuclei.

In this paper, we compared two different strategies. In the first approach, we performed the tumor/non-tumor classification of the cell nuclei taking advantage of the features extracted by the DL detection models. In the second case, quantitative descriptors of cell shape, morphology, and texture, i.e., pathomic features, are extracted using the PyRadiomics library [15], which offers a systematic pipeline for easing the quantitative analysis of medical images, with several applications in the radiomics field [16,17,18], but more recently for pathomics as well [19]. Both workflows are schematized in Figure 2.

The Mask R-CNN model played a key role in the detection stage. It is a two-stage detection method capable of tackling instance segmentation tasks [20] and is composed of two major building blocks: the backbone architecture, which performs feature extraction, and the head architecture, which performs classification, bounding box regression, and mask prediction [21]. We took advantage of the Detectron2 [22] implementation, which offers several models for object detection in PyTorch. To enable the adoption of the Detectron2 framework, the datasets’ annotations have been preliminarily converted into the COCO [23] annotation format.

The novel contribution concerning the proposed pipeline is twofold: (i) it has been integrated with the QuPath platform, allowing for a seamless integration with the pathologists’ workflow; and (ii) the analyzed pathomic feature set has been checked for consistency with clinical findings of nuclear characteristics to increase the trust of medical experts in adopting artificial intelligence-based CAD systems in their analyses.

#### 2.2.1. End-to-End Approach

In this approach, the classification of each cell nucleus as *tumor* or *non-tumor* directly occurs through the two-class (tumor/non-tumor) instance segmentation model. In this way, classification features are directly extracted by the DL model. We experimentally selected the model *mask_rcnn_R_50_C4_3x*. As can be seen from Table 2 and Table 3, this model achieved the largest average precision (AP) and average recall (AR) values; it has been trained with a Tesla T4, exploiting the Google Colab environment. Twenty thousand iterations were carried out in roughly 144 min. The chosen optimizer was SGDM, as set by default in the Detectron2 environment, selecting 0.00025 as the starting learning rate.

All models reported in Table 2 and Table 3 were evaluated on the internal validation data both qualitatively and quantitatively in order to identify the best performing architecture to use for experimentation on the external validation set. Once the best model was chosen, according to the quantitative metrics of Detectron2, a qualitative analysis was carried out by displaying the output of images coming from both the publicly available and the locally collected datasets.

#### 2.2.2. Two-Stage Approach

The classification phase is distinguished from the detection phase in the two-stage approach. Indeed, classification features are extracted through the PyRadiomics framework, allowing for the high-throughput feature extraction of pathomic characteristics from the nuclear regions. The rationale behind the considered pathomic features is that abnormalities of the appearance of a cell, such as shape, consistency, size, color, volume, and improper distribution of the nuclear-cytoplasmic ratio, are considered to be fundamental aspects in pathological evaluations [26,27].

The first step was to train a detection model with high accuracy for single-class instance segmentation. The *mask_rcnn_R_50_DC5_1x* model has been exploited according to previous evidence in this task [7]. The model was trained with a Tesla P100 by exploiting the Google Colab environment. Twenty thousand iterations were carried out in roughly 110 min. Also in this case, the chosen optimizer was SGDM, as set by default in the Detectron2 environment, which selected 0.00025 as the starting learning rate [7].

In the second phase, 83 features were extracted for each cell nucleus, exploiting the ground truth masks, and were used in order to train the classifier. Considered features include first-order statistics, 2D shape features, and texture features such as those extracted from the Gray Level Run Length Matrix (GLRLM) [28,29], Gray Level Co-Occurrence Matrix (GLCM) [30], Gray Level Size Zone Matrix (GLSZM) [31], and Gray Level Difference Matrix (GLDM) [32].

Features that showed a correlation coefficient larger than 0.8 were removed from the original set of 83 features, yielding a dataset of 29 features. Furthermore, to optimize the performance of the classifier, we chose an L1 penalty linear model, setting the regularization parameter C to 0.001 as the feature selection method and obtaining the final dataset of 16 features. Subsequently, the feature extraction and selection employed z-score normalization for the pre-processing of the data. Finally, an ML classifier capable of distinguishing between the two classes of nuclei was carefully devised. Both models based on support vector machines (SVMs) and artificial neural networks (ANNs) were compared. The details of the tried configurations are summarized in Table 4.

A performance evaluation was then carried out on both the publicly available and locally collected datasets.

#### 2.2.3. Evaluation Metrics

The comparison between detection models has been carried out through metrics that allow for a global evaluation, borrowing measures from the COCO evaluation system [23]. In detail, the following 10 metrics have been used to characterize the performance of an object detector: AP, $AP^{IoU=0.25}$, $AP^{IoU=0.50}$, $AP^{small}$, $AP^{medium}$, $AP^{large}$, $AR^{max=500}$, $AR^{small}$, $AR^{medium}$, and $AR^{large}$. These evaluation metrics are directly returned by the Detectron2 *COCOEvaluator* function. However, changes were made to the original function to return average precision results for several intersection over union (IoU) thresholds varying in the range [0.25, 0.75] (instead of the default range of [0.50, 0.95]). In addition, we replaced $AP^{IoU=0.50}$ and $AP^{IoU=0.75}$ with $AP^{IoU=0.25}$ and $AP^{IoU=0.50}$, respectively; for average recall, we used 500 detections per image instead of the default value of 100 (i.e., $AR^{max=500}$ instead of $AR^{max=100}$). Because H&E tissue tiles have a large number of nuclei instances, $AR^{max=1}$ and $AR^{max=10}$ were not included as quality measures in our analysis.

For the binary classification task for tumor/non-tumor nuclei, performances on the training and validation sets were evaluated by calculating the accuracy, F-Measure, and AUC-ROC (Area Under the Curve of Receiver Operating Characteristic).

For assessing the detection and classification results on the external validation ROIs, the considered quality metrics include precision, recall, and F-Measure results, as computed from object detection confusion matrices.

### 2.3. SHAP

The drawback of complex machine learning and deep learning classification models is that, despite their efficiency and accuracy, the rationale behind their decisions is unclear, leading to a lack of trust from expert physicians and patients.

The interpretability of a model decreases as its complexity increases. In recent years, different algorithms have been proposed to help explain which features contribute the most to the classification results, such as LIME [33] and SHAP [10]. The first algorithm learns an interpretable model locally around the prediction, whereas SHAP is based on the optimal Shapley values from the game theory. In this work, we focused on SHAP as previous findings have reported that it provides better results for the task and the models considered in this study [34].

SHAP values are calculated by determining the average of the difference between all possible combinations of predicted values, considering them with and without each feature. Thus, it becomes possible to understand the magnitude and sign of feature importance by calculating the SHAP values. Feature impact is defined as the change it provokes in the model output compared with when that feature is not considered. Given a specific model *f*, the Shapley values $\phi_{i}$ for the features i∈F are calculated using a weighted sum that represents the impact of each feature being added to the model, which is scaled by the number of all possible orders of features being introduced [10], as shown in Equation (1).

(1) $$\phi_{i}=\sum_{S\subseteq F\setminus \{i\}}\frac{|S|!(|F|-|S|-1)!}{|F|!}\left[f_{S\cup \{i\}}\left(x_{S\cup \{i\}}\right)-f_{S}\left(x_{S}\right)\right]$$

Iteration takes place on all possible subsets S⊆F, where *F* is the set of all features, and for each of them, a new model is trained. To compute the effect of including a feature on the model prediction, a model $f_{S\cup \{i\}}$ is trained with that feature present, and another model $f_{S}$ is trained with the feature withheld. $x_{S}$ represents the values of the input features in set *S*.

To explain the output of a neural network model, we used the Kernel SHAP method [10], offered by the class *KernelExplainer* of the Python package SHAP [35]; this method uses a special weighted linear regression to compute the impact of each feature. The computed importance values are Shapley values from game theory [36] together with local linear regression coefficients.

The *KernelExplainer* does not sample from the training dataset automatically; it attempts to use the entire dataset. In order to speed up the calculations, we reduced the size of the training set using a small portion of it; in order to obtain such a reduction, a clustering algorithm can be used, or *k* data instances can be randomly sampled [37]. In our experiments, the training dataset was clustered using *k*-means, choosing k=100. Cluster weights are proportional to the number of data points in each cluster. Our training dataset was broken down into clusters, and weighted mean values were calculated for each cluster. The SHAP values are calculated based on this weighted dataset. We have to identify a proper quantity of samples in order to determine the SHAP values by resampling the training dataset and evaluating the impact over these perturbations; therefore, in our study, we employed a total of 500 samples and determined the feature impact on the test set [37]. During the experiment, we exploited the Python package SHAP, which provides various tools for displaying the Shapley values and identifies the most important features (summary plot, waterfall plot, decision plot, and force plot).

### 2.4. Integration with QuPath

**Figure 3:**
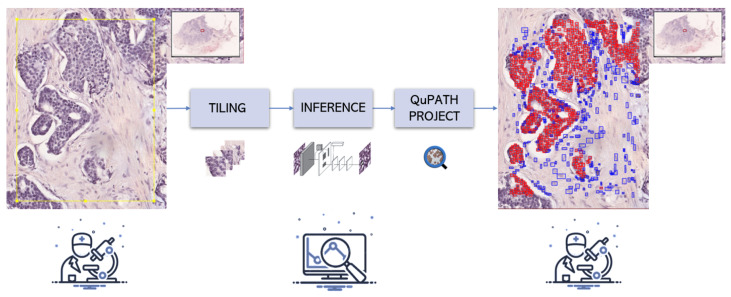
QuPath integration workflow. First, an ROI is selected by the operator. Then, the ROI is divided into smaller chunks that can be fed to a deep learning algorithm. Afterward, the results are reconstructed at the WSI-level and saved into a QuPath project to allow for visualization inside the QuPath environment.

One interesting aspect of the proposed workflow concerns the integration of a deep learning model with the QuPath Software to facilitate pathologists in their WSI analyses. Indeed, the first operation consists of the operator annotating an ROI on the QuPath interface. Then, the ROI is divided into tiles to permit the inference of the detectors on small chunks of the WSI. Bounding boxes are saved for each cell nucleus, together with the class label (tumor or non-tumor). Then, all predictions are reconstructed on the original WSI, considering the proper offset of the specific tile. A QuPath project with annotations can be finally built by exploiting PAQUO (PAthological QUpath Obsession) [38], a Python library for interacting with QuPath. The overall procedure is portrayed in Figure 3.

## 3. Results

### 3.1. Instance Segmentation

Detection results in terms of the measures introduced in Section 2.2.3 are reported in Table 3 for measures related to AP and Table 2 for measures linked to AR and for both the one-class instance segmentation (D1C) and two-class instance segmentation (D2C) models. D1C has been assessed in terms of average precision, considering only the nucleus class; hence, its results have to be seen in conjunction with the subsequent classification results. D2C has been assessed by considering both the tumor nucleus and the non-tumor nucleus classes.

**Figure 4:**
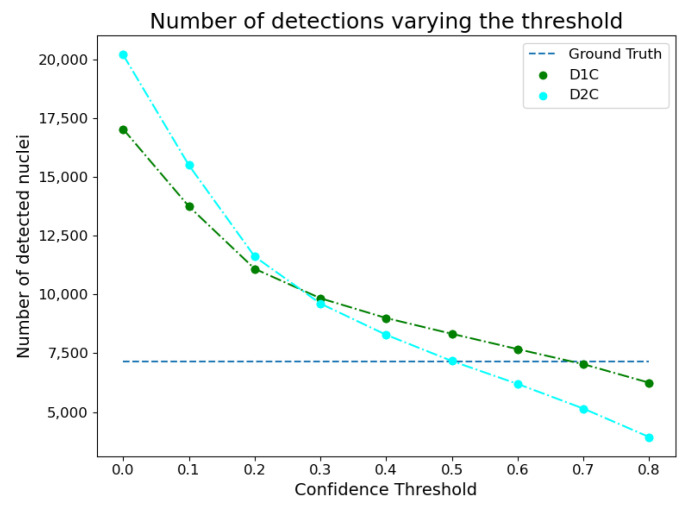
Experimental analysis for finding the best confidence threshold.

**Figure 5:**
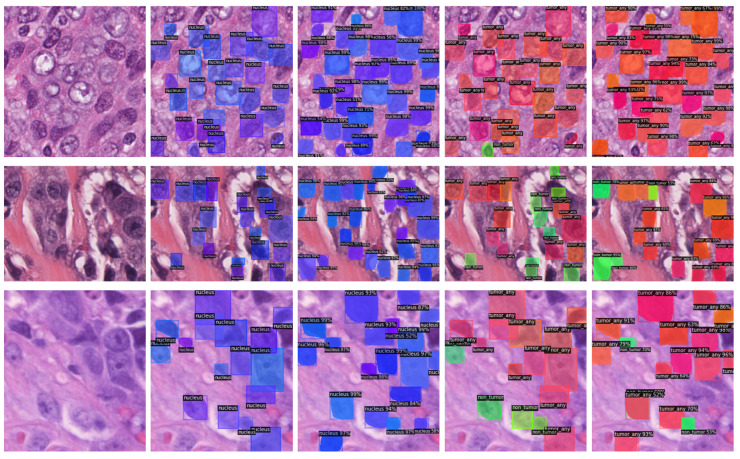
Internal validation. (**First Column**) Original images. (**Second Column**) Ground truth for the D1C approach. (**Third Column**) Model predictions for the D1C approach. (**Fourth Column**) Ground truth for the D2C approach. (**Fifth Column**) Model predictions for the D2C approach. For the D1C approach, blue represents the nucleus class; for the D2C approach, green and red represent the non-tumor nucleus and tumor nucleus classes, respectively.

In order to select the most appropriate confidence threshold to be used in the instance segmentation models, the number of detections has been compared with the ground truth for different values of the confidence threshold. Figure 4 shows that setting the threshold to 0.5 was a reasonable choice. Indeed, with this threshold value, both approaches detected at least as many nuclei as those in the ground truth. A higher number of detections can be spotted for the D1C model, but this is justified by the fact that the model detects more nuclei with respect to the ground truth. Indeed, in some cases, not all nuclear instances may be annotated. Examples of such detections can be seen in Figure 5.

For exploiting the D1C model in the combined task of nuclei detection and classification, a binary classifier has been added to discriminate between tumor and non-tumor nuclei. The performances obtained by the binary classification models described in Table 4, trained on features extracted with the PyRadiomics framework, are reported in Table 5. The resulting best model is the ANN method with ID = 4 as it has the highest AUC on both the training and test sets and is the one with the least number of neurons.

**Figure 6:**
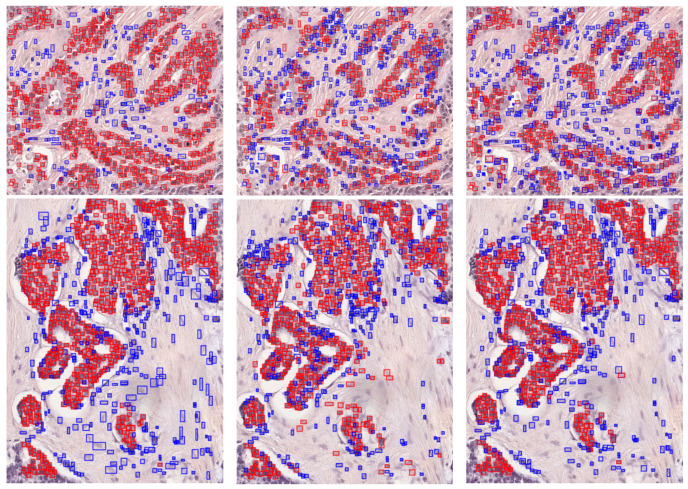
External validation visualization examples in QuPath. (**First column**) Ground truth. (**Second column**) D1C model. (**Third column**) D2C model. Blue: non-tumor nuclei; red: tumor nuclei.

For the external validation phase, the metrics were extracted from both the D1C and D2C workflows, starting from the object detection confusion matrices obtained for each of the two ROIs. The comparison was based on the prediction obtained from the proposed approach and the ground truth generated by the expert pathologists. Figure 6 portrays the detection results on these two ROIs. Quantitative results of the external validation phase are summarized in Table 6.

### 3.2. Pathomic Features and SHAP Analysis

**Figure 7:**
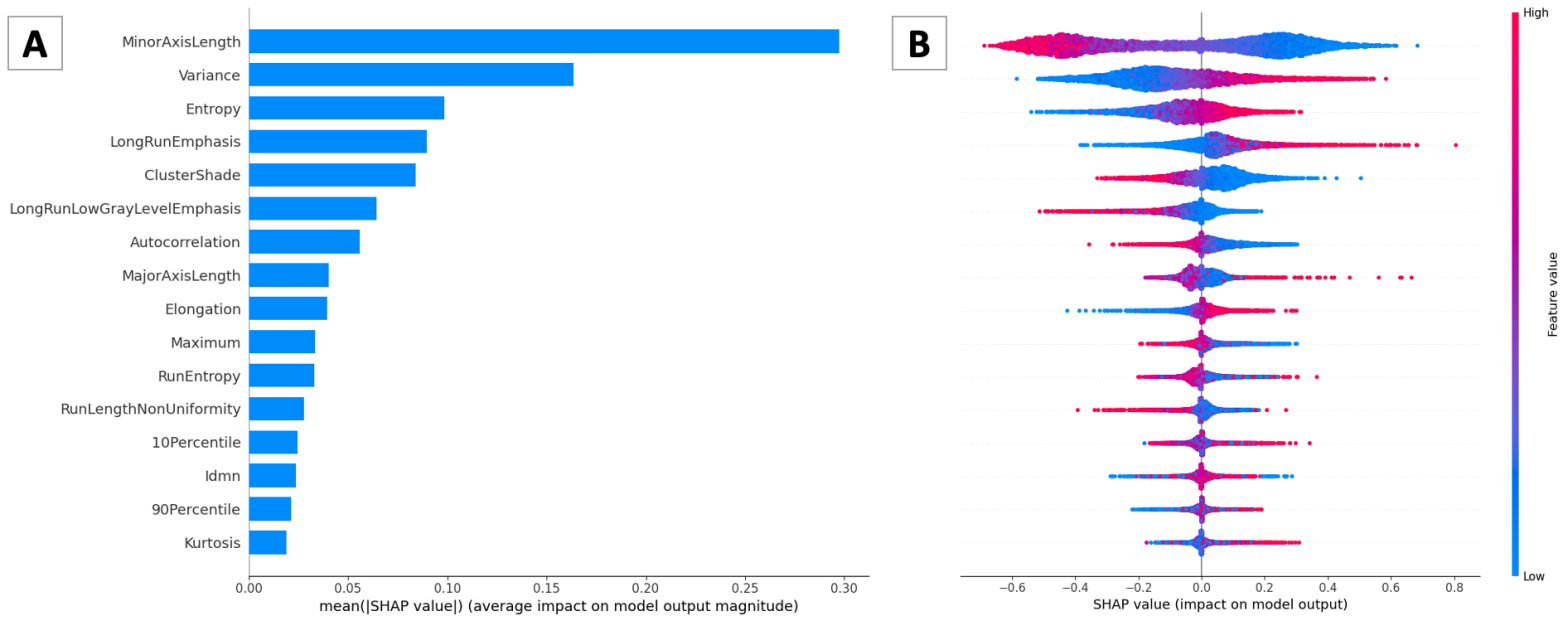
(**A**) **Bar plot**. The plot depicts the average impact of the considered features according to the magnitude of the SHAP values. The most important feature is *MinorAxisLength*. (**B**) **Summary plot**. The plot displays the fusion between feature importance and feature effects by plotting a point for each Shapley value belonging to a feature and an instance. Higher values of *MinorAxisLength* are associated with a greater risk of being a tumor nucleus.

To show the contribution or importance of each feature on the ANN predictions, different charts portraying the obtained SHAP values have been realized. As the first step, we determined the most important features by plotting them with decreasing SHAP impact, as reported in Figure 7.

Features are ordered from the most to the least important. We can see that *MinorAxisLength* is the most important feature. Generally, the greater the value of this feature, the more likely the target will be a tumor nucleus. The lower the value of this feature, the less likely the target will be a tumor nucleus. The average impact of these features according to the magnitude of SHAP values is displayed in Figure 7A, which depicts the bar plot. Even though this plot does not depict the sign of the contributions, it allows us to have an idea of the magnitude of the features’ importance.

The feature importance and feature effects are fused in the summary plot, as portrayed in Figure 7B. Every point displayed on this plot is associated with a Shapley value belonging to a feature and instance. Locations of points are dependent on the feature name for the y-axis and on the Shapley value for the x-axis. The color of the points indicates the feature value, ranging from low values to high ones. To avoid overlapping points, jittering is applied on the y-axis for nearby points. This helps in understanding the Shapley values’ distribution for each feature [39].

**Figure 8:**
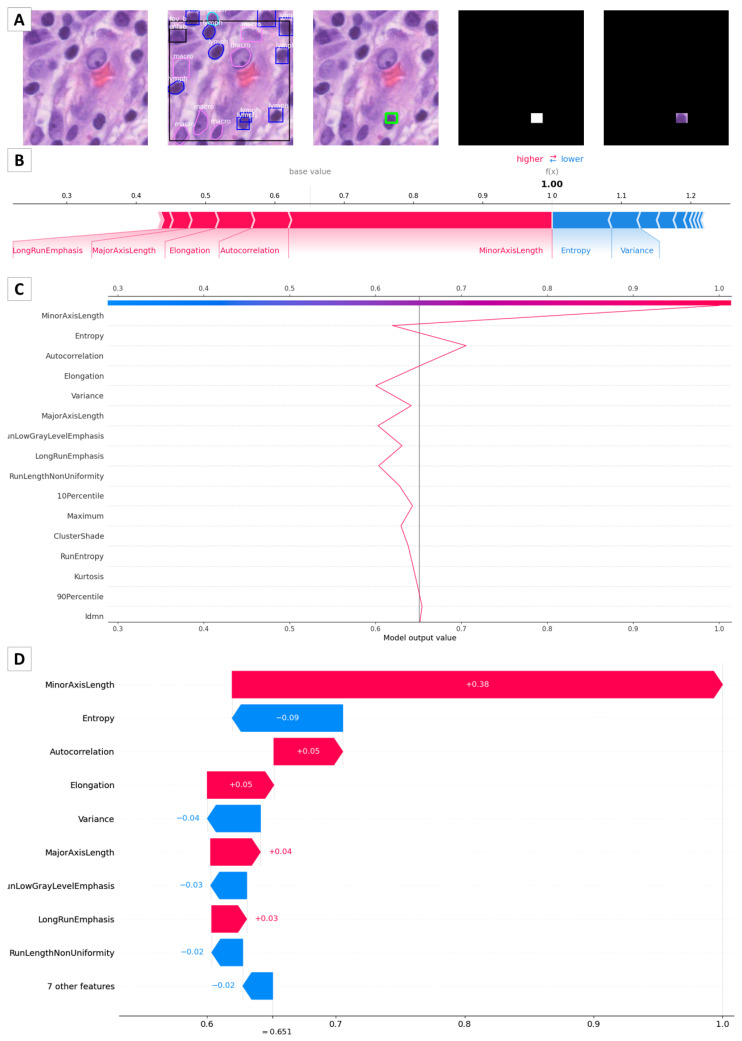
(**A**) **Example nucleus**. The first row depicts a lymphocytic nucleus that has been considered as a sample on which to perform the investigation of feature importance analysis. (**B**) **Force plot**. The stripes in the figure demonstrate the feature impact with respect to the target variable. In detail, the red and blue stripes display the features increasing and decreasing the base value, respectively. (**C**) **Decision plot**. The plot depicts the decision graph, which allows for the inspection of the magnitude of each decision change according to the features. (**D**) **Waterfall plot**. The plot portrays an outlook of the magnitude and sign of the impact of each feature.

Figure 8 portrays different plots that can be used to explain to physicians, patients, and other stakeholders the features’ contribution to the final model prediction. The considered nucleus for this example is reported in Figure 8A. Then, Figure 8B portrays the force plot. For this sample, the predicted value (expected value) is one, corresponding to the non-tumor class. The base value is computed as the mean value of the target variable on all samples. The stripes in the figure demonstrate the feature impact with respect to the target variable. In detail, the red (blue) stripes display the features that increase (or decrease) the base value. A larger strip is associated with a higher (as absolute value) feature contribution. All contributions are summed to obtain the final expected value for the target variable [40]. As we can see, for this sample, the features *MinorAxisLength*, *Autocorrelation*, *Elongation*, *MajorAxisLength*, and *LongRunEmphasis* have a positive contribution to the expected value. *MinorAxisLength* is still the most important descriptor in this example because its contribution is the largest one (it has the largest streak). On the other hand, *Entropy*, *Variance*, and some other less important features have a negative contribution. In the end, the total positive contribution (red stripes) is greater than the negative contribution (blue stripes); hence, the final value is greater than the base value. Figure 8C,D depict the decision graph, which permits us to observe the magnitude of each change, and the waterfall chart, which allows us to examine the magnitude and sign of the impact of the features. Considering all of these graphs, the user can have a clear view of which features contribute the most and how they are affecting the decision-making process.

**Figure 9:**
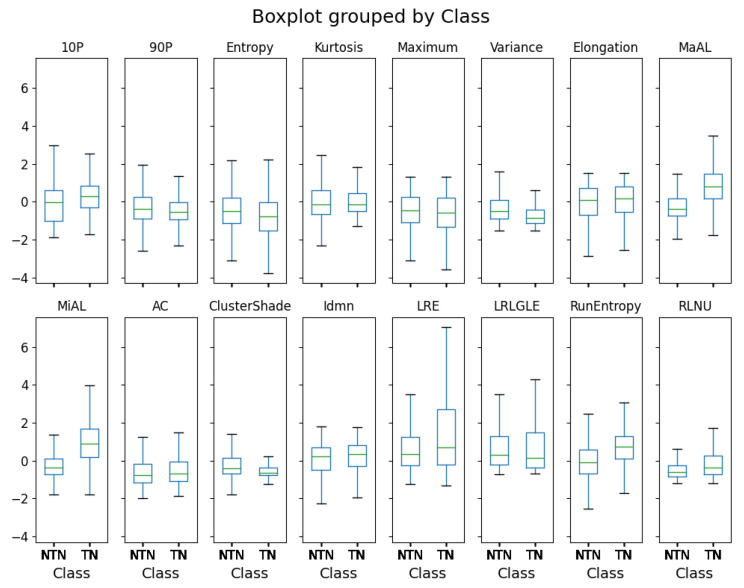
Boxplots for the selected features. NTN stands for non-tumor nucleus; TN stands for tumor nucleus; 10P stands for *10Percentile*; 90P stands for *90Percentile*; MaAL stands for *MajorAxisLength*; MiAL stands for *MinorAxisLength*; LRLGLE stands for *LongRunLowGrayLevelEmphasis*; RLNU stands for *RunLengthNonUniformity*; AC stands for *Autocorrelation*; LRE stands for *LongRunEmphasis*. All of the selected features show statistically significant differences according to the Mann–Whitney U test.

In order to complement the views offered by SHAP, Figure 9 reveals the feature values distribution. All 16 selected features are statistically significant according to the Mann–Whitney U test, with the significance threshold being set to 0.05. Kurtosis presents a *p*-value of 0.04. All other features display *p*-values that are less than 0.001.

## 4. Discussion

During our experiments, two strategies of cell nuclei classification were tested. Both approaches were based on machine learning and deep learning models, including instance segmentation architectures and artificial neural networks.

The challenges in cellular nuclei detection and classification include that nuclei are very small and are often close or overlapped; hence, semantic segmentation algorithms may fail if not used in conjunction with particular strategies or post-processing operations [7,41,42]. Indeed, this work considers an instance segmentation approach for the first stage of individuating the nuclear regions. Then, one of the two proposed workflows performs the classification of nuclei as tumor/non-tumor by exploiting quantitative descriptors, i.e., pathomic features, which take into account the morphological and textural characteristics of nuclei. Quantitative results are discussed in Section 4.1, whereas a clinical perspective, in which the considered pathomic features have been validated by an expert pathologist, is reported in Section 4.2.

### 4.1. Instance Segmentation and Classification

The first approach herein analyzed consists of an end-to-end detection and classification as carried out by the Mask R-CNN instance segmentation model. The analysis of the COCO metrics on the internal validation dataset shows an AP of 54.0% and an $AP^{IoU=0.25}$ equal to 68.8%. These values are acceptable considering the complexity of the task, as shown by the comparison between different versions of Mask R-CNN in Table 3 and Table 2. With a confidence threshold set to 0.5, the number of detected instances is 7166 compared with 7150 instances of ground truth, indicating that the model predictions are comparable in number with the ground truth annotations. Furthermore, the model also detects instances in correspondence of non-annotated nuclei, or of darker areas due to slide artifacts.

The second approach consists of two separate steps for the detection task, which was performed by D1C, and for the classification task, which was performed by the machine learning methods processing the pathomic features. Mask R-CNN detects a number of instances equal to 8322 with a confidence threshold setting of 0.5. In this case, an over-detection can be noticed. This is due to the fact that the model also detects nuclei that were not annotated in the dataset. In the internal validation set, the model showed an AP value of 72.2% and an $AP^{IoU=0.25}$ value of 89.1%. Graphical visualization of the output super-imposed on the images highlights that almost all the instances have been identified; however, we can notice cases in which the model detects nuclei not appearing in the ground truth and vice versa.

The second phase of this second approach involves the training of a supervised learning model capable of classifying each nucleus as “tumor” or “non-tumor”, by analyzing the pathomic features that describe morphological, shape, statistical, and textural information. Indeed, large, irregularly shaped, fragmented, and discolored nuclei are the signs of the presence of a malignant tumor [43]. Seven different classifiers were trained, and among these, the best performances were obtained while using an ANN, as reported in Table 5. In fact, this model achieved an Accuracy of 77%, an F1-Score of 80%, and an AUC equal to 86% on the test set.

The final comparison between the two models involves the external validation phase, in which the entire workflow was tested, including integration with the QuPath software. Both models have been tested on the two ROIs extracted from the WSIs, and thanks to the confusion matrix for object detection and relative indices, it is possible to understand what the general performance of the two approaches are. The first model demonstrates an excellent ability for detecting and recognizing the tumor nuclei, whereas it has a slightly worse performance for the non-tumor nuclei, which the model tends to confuse with tumor nuclei. In fact, the values of precision, recall, and F-measure are very high for tumor nuclei and lower for non-tumor nuclei. The same trend occurs in the case of the second approach, for which, however, performance indices are generally lower.

Evaluating only the task of detection, the Mask R-CNN model trained only on one class shows a very promising performance; however, adding a classifier on top of this detector reduces the performance of the combined detection and classification task. Hence, from an accuracy perspective, the end-to-end approach is the one with the best performance.

### 4.2. Clinical Perspective

The detection and classification of nuclei is a challenging task that, in the clinical routine, relies on the eye scale of pathologists. In the era of precision medicine, and thus the setting of therapeutic approaches based on tumor molecular alterations, the percentage of neoplastic cellularity has to be estimated to correctly identify them. Indeed, molecular biologists dissect areas with the greatest percentage of tumor nuclei to measure the expression of specific signatures to predict prognosis (e.g., EndoPredict or MammaPrint [44]). The availability of an automatic tool that can aid pathologists and molecular biologists in accurately measuring tumor cellularity can greatly improve the prognostic reliability of tests conducted on cancer patients. On the other hand, to ease the adoption in clinical practice, medical experts need to have an understanding of how the model is processing the input features.

The proposed two-stage approach allows for a closer understanding of the characteristics that are involved in classifying nuclei as tumoral or non-tumoral by an artificial neural network. Indeed, a very interesting aspect of this work is the analysis conducted with SHAP to understand the relevance of the features. In the example considered in Figure 8, the features that most positively contribute to the model classification are *MinorAxisLength*, *Autocorrelation*, *Elongation*, and *MajorAxisLength*.

Among them, three are part of the shape features, and the remaining one is derived from the GLCM, which is fundamental for the texture analysis. This result confirms the histopathological findings of tumor nuclei, which generally report morphological abnormalities that make it possible to distinguish between healthy and malignant cells. In fact, in the case of tumor nuclei, nuclear enlargement occurs, as well as an increase in the nucleus–cytoplasm ratio, irregularity of the nuclear membrane, hyperchromasia, and anomalous distribution of chromatin.

Features regarding the shape of nuclei, notwithstanding the great variability between the several cytotypes, are also the most effective in the evaluation performed by pathologists. Interestingly, Reza et al. [45] demonstrated that object-level features, including *MajorAxisLength*, achieved better results than spatial arrangement features when performing tumor grading, which is a classification linked to the aggressiveness and invasion of the tumor, as performed from nuclei in the glioma TCGA cohort. Nevertheless, we note that the length of the minor axis may be a better estimator of nuclear size and therefore the most effective discriminator between normal and tumor nuclei, as it is less affected than the major axis length by the angle of incidence of the section plane [46].

Moreover, Figure 8 displays, for the example nucleus under consideration, an important negative contribution of *Entropy*, which is the second most important feature after *MinorAxisLength*, and *Variance*, which is the fifth most significant feature. According to Figure 7, *Variance* end *Entropy* have negative contributions to the model classification in most cases. This means that lower values of these features are generally associated with tumor nuclei. Instead, higher values of these features are generally linked to non-tumor nuclei. The lesser extent of *Variance* end *Entropy* in tumor nuclei may be due to their clonal nature, thus showing a pattern of biological and metabolic pathways that are very similar to each other with respect to the several cell populations present in the tumor microenvironment.

The hyperchromatic feature of tumor nuclei, which represents the phenotypic expression of genomic instability and aberrant gene expression, is well-known. In particular, breast cancer is a family of diseases and, among the other features, hyperchromasia has been observed and compared for the purposes of differentiating the several subtypes [47,48], confirming its importance. Hyperchromasia has the effect of a paint dab, as confirmed by pathologists, reducing the variability in the texture. Hence, this can be another reason for the negative contribution of *Variance* end *Entropy* to the model classification.

## 5. Conclusions and Future Works

Detection and classification of nuclei of cells are among the most important operations in digital pathology workflows. In this work, we considered two approaches for this aim: an end-to-end method, which directly exploited an instance segmentation framework to perform nuclei detection and classification; and a two-stage method, in which ML classifiers are trained for recognizing tumor nuclei on top of pathomic features, which describe the quantitative characteristics of nuclei. The first method shows the highest detection and classification combined results. However, the pathomic features analyzed in the two-step pipeline can allow for the achievement of a better understanding of the characteristics as considered by artificial neural networks to perform such classifications, creating a trust for clinicians and patients in AI-powered CAD systems.

Future works will involve the collection of annotated datasets from other organs and tissues in order to have a broader validation of the developed models. Furthermore, other deep learning architectures and XAI techniques can be added to the analysis framework in order to realize ever more accurate, efficient, and explainable CAD systems for digital pathology scenarios.

## Figures and Tables

**Table 1:** Details of the considered datasets. For the NuCLS dataset, *sTILs* denotes stromal tumor-infiltrating lymphocytes. Only the super-classes of the NuCLS dataset have been reported.

Dataset	Publication Year	Organ	Magnification	Annotated Nuclei	Classes
NuCLS [13]	2019	Breast	40×	59,485	Tumor, Stromal, sTILs, Other, Ambiguous
Local [12]	2023	Breast	40×	2024	Tumor, Non-tumor

**Table 2:** ML classifiers (implementation details). Parameters’ names are reported according to the *scikit-learn* documentation [24].

	ID	C	Kernel			
**SVM**	1	5	rbf			
	2	1	rbf			
	3	50	poly			
	**ID**	**Hidden Layers**	**Solver**	**Learning Rate**	**Activation**	**Max Iter**
**ANN**	4	(100)	adam	constant	relu	500
	5	(150)	sgd	adaptive	relu	500
	6	(80, 80)	sgd	adaptive	logistic	1000
	7	(100, 80)	adam	constant	logistic	1500

**Table 3:** Detection model performances based on AP. Architecture refers to the Mask R-CNN configurations available in the Detectron2 COCO-InstanceSegmentation [25].

Approach	Architecture	AP	${\mathrm{AP}}^{\mathrm{IoU}=0.25}$	${\mathrm{AP}}^{\mathrm{IoU}=0.50}$	${\mathrm{AP}}^{\mathrm{small}}$	${\mathrm{AP}}^{\mathrm{medium}}$	${\mathrm{AP}}^{\mathrm{large}}$
**D1C**	**R_50_DC5_1x**	**0.722**	**0.891**	**0.803**	**0.711**	**0.739**	**0.527**
**D2C**	R_50_DC5_1x	0.519	0.651	0.584	0.380	0.471	**0.550**
	R_50_DC5_3x	0.533	0.682	0.603	0.387	0.482	0.321
	R_50_FPN_1x	0.493	0.628	0.556	0.374	0.447	0.320
	**R_50_C4_3x**	**0.540**	**0.688**	0.603	**0.428**	0.473	0.413
	R_101_DC5_3x	0.536	0.686	**0.606**	0.389	**0.495**	**0.550**
	X_101_32x8d_FPN_3x	0.483	0.634	0.543	0.339	0.433	**0.550**

**Table 4:** Detection model performances based on AR. Architecture refers to the Mask R-CNN configurations available in the Detectron2 COCO-InstanceSegmentation [25].

Approach	Architecture	${\mathrm{AR}}^{\max=500}$	${\mathrm{AR}}^{\mathrm{small}}$	${\mathrm{AR}}^{\mathrm{medium}}$	${\mathrm{AR}}^{\mathrm{large}}$
**D1C**	**R_50_DC5_1x**	**0.839**	**0.820**	**0.858**	**0.545**
**D2C**	R_50_DC5_1x	0.777	0.699	0.746	**0.545**
	R_50_DC5_3x	0.781	0.678	0.779	0.318
	R_50_FPN_1x	0.758	0.696	0.723	0.364
	**R_50_C4_3x**	**0.798**	**0.718**	**0.802**	0.409
	R_101_DC5_3x	0.783	0.711	0.768	**0.545**
	X_101_32x8d_FPN_3x	0.710	0.645	0.663	**0.545**

**Table 5:** Binary classification models results.

Model	ID	Accuracy	F-Measure	AUC	Accuracy	F-Measure	AUC
		(Train)	(Train)	(Train)	(Test)	(Test)	(Test)
**SVM**	1	0.83	0.87	0.89	0.77	0.80	0.84
	2	0.83	0.87	0.89	0.77	0.81	0.85
	3	0.82	0.87	0.89	0.77	0.80	0.82
**ANN**	4	0.83	0.87	0.90	0.77	0.80	0.86
	5	0.82	0.86	0.89	0.78	0.81	0.86
	6	0.81	0.86	0.88	0.78	0.81	0.84
	7	0.83	0.87	0.90	0.77	0.80	0.85

**Table 6:** Metrics on the external validation ROIs.

ROI	Approach	Tumor Nucleus			Non-Tumor Nucleus		
		Precision	Recall	F-Measure	Precision	Recall	F-Measure
1	D2C	0.963	0.954	0.958	0.839	0.864	0.851
2	D2C	0.897	0.726	0.802	0.417	0.964	0.582
1	D1C	0.854	0.714	0.778	0.524	0.522	0.523
2	D1C	0.857	0.578	0.691	0.343	0.665	0.453

## Data Availability

The NuCLS [13] dataset is publicly available. The local dataset from IRCCS Istituto Tumori “Giovanni Paolo II” presented in this study has been made publicly available [12].

## References

[1] Rodriguez J.P.M., Rodriguez R., Silva V.W.K., Kitamura F.C., Corradi G.C.A., de Marchi A.C.B., Rieder R. (2022). Artificial intelligence as a tool for diagnosis in digital pathology whole slide images: A systematic review. J. Pathol. Inform..

[2] Gupta R., Kurc T., Sharma A., Almeida J.S., Saltz J. (2019). The emergence of pathomics. Curr. Pathobiol. Rep..

[3] Manivannan S., Li W., Akbar S., Wang R., Zhang J., McKenna S.J. (2016). An automated pattern recognition system for classifying indirect immunofluorescence images of HEp-2 cells and specimens. Pattern Recognit..

[4] Zheng Y., Jiang Z., Xie F., Zhang H., Ma Y., Shi H., Zhao Y. (2017). Feature extraction from histopathological images based on nucleus-guided convolutional neural network for breast lesion classification. Pattern Recognit..

[5] Van der Velden B.H., Kuijf H.J., Gilhuijs K.G., Viergever M.A. (2022). Explainable artificial intelligence (XAI) in deep learning-based medical image analysis. Med. Image Anal..

[6] Hussain S.M., Buongiorno D., Altini N., Berloco F., Prencipe B., Moschetta M., Bevilacqua V., Brunetti A. (2022). Shape-Based Breast Lesion Classification Using Digital Tomosynthesis Images: The Role of Explainable Artificial Intelligence. Appl. Sci..

[7] Altini N., Brunetti A., Puro E., Taccogna M.G., Saponaro C., Zito F.A., De Summa S., Bevilacqua V. (2022). NDG-CAM: Nuclei Detection in Histopathology Images with Semantic Segmentation Networks and Grad-CAM. Bioengineering.

[8] Altini N., Marvulli T.M., Caputo M., Mattioli E., Prencipe B., Cascarano G.D., Brunetti A., Tommasi S., Bevilacqua V., Summa S.D. Multi-class Tissue Classification in Colorectal Cancer with Handcrafted and Deep Features. Proceedings of the International Conference on Intelligent Computing.

[9] Ploug T., Holm S. (2020). The four dimensions of contestable AI diagnostics-A patient-centric approach to explainable AI. Artif. Intell. Med..

[10] Lundberg S.M., Lee S.I. A unified approach to interpreting model predictions. Proceedings of the Advances in Neural Information Processing Systems.

[11] Bankhead P., Loughrey M.B., Fernández J.A., Dombrowski Y., McArt D.G., Dunne P.D., McQuaid S., Gray R.T., Murray L.J., Coleman H.G. (2017). QuPath: Open source software for digital pathology image analysis. Sci. Rep..

[12] Altini N., Puro E., Taccogna M.G., Marino F., De Summa S., Saponaro C., Mattioli E., Zito F.A., Bevilacqua V. (2023). A Dataset of Annotated Histopathological Images for Tumor Cellularity Assessment in Breast Cancer.

[13] Amgad M., Elfandy H., Hussein H., Atteya L.A., Elsebaie M.A., Abo Elnasr L.S., Sakr R.A., Salem H.S., Ismail A.F., Saad A.M. (2019). Structured crowdsourcing enables convolutional segmentation of histology images. Bioinformatics.

[14] Weinstein J., Collisson E., Mills G., Shaw K.M., Ozenberger B., Ellrott K., Shmulevich I., Sander C., Stuart J., The Cancer Genome Atlas Research Network (2013). The cancer genome atlas pan-cancer analysis project. Nat. Genet..

[15] Van Griethuysen J.J., Fedorov A., Parmar C., Hosny A., Aucoin N., Narayan V., Beets-Tan R.G., Fillion-Robin J.C., Pieper S., Aerts H.J. (2017). Computational radiomics system to decode the radiographic phenotype. Cancer Res..

[16] Laukamp K.R., Shakirin G., Baeßler B., Thiele F., Zopfs D., Hokamp N.G., Timmer M., Kabbasch C., Perkuhn M., Borggrefe J. (2019). Accuracy of radiomics-based feature analysis on multiparametric magnetic resonance images for noninvasive meningioma grading. World Neurosurg..

[17] Bevilacqua V., Altini N., Prencipe B., Brunetti A., Villani L., Sacco A., Morelli C., Ciaccia M., Scardapane A. (2021). Lung Segmentation and Characterization in COVID-19 Patients for Assessing Pulmonary Thromboembolism: An Approach Based on Deep Learning and Radiomics. Electronics.

[18] Brunetti A., Altini N., Buongiorno D., Garolla E., Corallo F., Gravina M., Bevilacqua V., Prencipe B. (2022). A Machine Learning and Radiomics Approach in Lung Cancer for Predicting Histological Subtype. Appl. Sci..

[19] Knabbe J., Das Gupta A., Kuner T., Asan L., Beretta C., John J. (2022). Comprehensive monitoring of tissue composition using in vivo imaging of cell nuclei and deep learning. bioRxiv.

[20] Du L., Zhang R., Wang X. (2020). Overview of two-stage object detection algorithms. Proceedings of the Journal of Physics: Conference Series.

[21] He K., Gkioxari G., Dollár P., Girshick R. Mask r-cnn. Proceedings of the IEEE International Conference on Computer Vision.

[22] Wu Y., Kirillov A., Massa F., Lo W.Y., Girshick R. (2019). Detectron2. https://github.com/facebookresearch/detectron2.

[23] Lin T.Y., Maire M., Belongie S., Hays J., Perona P., Ramanan D., Dollár P., Zitnick C.L. Microsoft coco: Common objects in context. Proceedings of the Computer Vision—ECCV 2014: 13th European Conference.

[24] Scikit-Learn Machine Learning in Python. https://scikit-learn.org/stable/.

[25] Detectron2 COCO-InstanceSegmentation. https://github.com/facebookresearch/detectron2/tree/main/configs/COCO-InstanceSegmentation.

[26] Irshad H., Veillard A., Roux L., Racoceanu D. (2013). Methods for nuclei detection, segmentation, and classification in digital histopathology: A review—current status and future potential. IEEE Rev. Biomed. Eng..

[27] Ra˛czkowski Ł., Moz˙ ejko M., Zambonelli J., Szczurek E. (2019). ARA: Accurate, reliable and active histopathological image classification framework with Bayesian deep learning. Sci. Rep..

[28] Galloway M.M. (1975). Texture analysis using gray level run lengths. Comput. Graph. Image Process..

[29] Chu A., Sehgal C.M., Greenleaf J.F. (1990). Use of gray value distribution of run lengths for texture analysis. Pattern Recognit. Lett..

[30] Haralick R.M., Dinstein I., Shanmugam K. (1973). Textural Features for Image Classification. IEEE Trans. Syst. Man Cybern..

[31] Thibault G., Fertil B., Navarro C., Pereira S., Cau P., Levy N., Sequeira J., Mari J.l. Texture Indexes and Gray Level Size Zone Matrix Application to Cell Nuclei Classification. Proceedings of the 10th International Conference on Pattern Recognition and Information Processing, PRIP 2009.

[32] Sun C., Wee W.G. (1983). Neighboring gray level dependence matrix for texture classification. Comput. Vision Graph. Image Process..

[33] Ribeiro M.T., Singh S., Guestrin C. “Why should i trust you?” Explaining the predictions of any classifier. Proceedings of the 22nd ACM SIGKDD International Conference on Knowledge Discovery and Data Mining.

[34] Hailemariam Y., Yazdinejad A., Parizi R.M., Srivastava G., Dehghantanha A. An empirical evaluation of AI deep explainable tools. Proceedings of the 2020 IEEE Globecom Workshops (GC Wkshps).

[35] SHAP Documentation. https://shap.readthedocs.io/en/latest/index.html.

[36] Rozemberczki B., Watson L., Bayer P., Yang H.T., Kiss O., Nilsson S., Sarkar R. (2022). The shapley value in machine learning. arXiv.

[37] Bagheri R. Introduction to SHAP Values and Their Application in Machine Learning. https://towardsdatascience.com/introduction-to-shap-values-and-their-application-in-machine-learning-8003718e6827.

[38] PAQUO Documentation. https://paquo.readthedocs.io/en/latest/index.html..

[39] Molnar C. (2022). Interpretable Machine Learning: A Guide for Making Black Box Models Explainable.

[40] Malato G. How to Explain Neural Networks Using SHAP. https://www.yourdatateacher.com/2021/05/17/how-to-explain-neural-networks-using-shap/.

[41] Altini N., Cascarano G.D., Brunetti A., Marino F., Rocchetti M.T., Matino S., Venere U., Rossini M., Pesce F., Gesualdo L. (2020). Semantic segmentation framework for glomeruli detection and classification in kidney histological sections. Electronics.

[42] Altini N., Cascarano G.D., Brunetti A., De Feudis I., Buongiorno D., Rossini M., Pesce F., Gesualdo L., Bevilacqua V. (2020). A deep learning instance segmentation approach for global glomerulosclerosis assessment in donor kidney biopsies. Electronics.

[43] Tripathi S., Singh S.K. (2020). Ensembling handcrafted features with deep features: An analytical study for classification of routine colon cancer histopathological nuclei images. Multimed. Tools Appl..

[44] Jahn S.W., Bösl A., Tsybrovskyy O., Gruber-Rossipal C., Helfgott R., Fitzal F., Knauer M., Balic M., Jasarevic Z., Offner F. (2020). Clinically high-risk breast cancer displays markedly discordant molecular risk predictions between the MammaPrint and EndoPredict tests. Br. J. Cancer.

[45] Reza S.M., Iftekharuddin K.M. Glioma grading using cell nuclei morphologic features in digital pathology images. Proceedings of the Medical Imaging 2016: Computer-Aided Diagnosis.

[46] Fischer E.G. (2020). Nuclear morphology and the biology of cancer cells. Acta Cytol..

[47] Yeom C., Kim H., Kwon S., Kang S.H. (2016). Clinicopathologic Features of Pleomorphic Invasive Lobular Carcinoma: Comparison with Classic Invasive Lobular Carcinoma. J. Breast Dis..

[48] Ishitha G., Manipadam M.T., BackIanathan S., Chacko R.T., Abraham D.T., Jacob P.M. (2016). Clinicopathological study of triple negative breast cancers. J. Clin. Diagn. Res..

